# VNS improves VSMC metabolism and arteriogenesis in infarcted hearts through m/n-AChR-Akt-SDF-1α in adult male rats

**DOI:** 10.1007/s10735-023-10171-4

**Published:** 2024-01-02

**Authors:** Xing-yuan Li, Jia-Qi Liu, Yan Wang, Yan Chen, Wen-hui Hu, Yan-xia Lv, Yan Wu, Jing Lv, Jun-ming Tang, Deying Kong

**Affiliations:** 1 Department of Physiology, Faculty of Basic Medical Sciences, Zunyi Medicical University, Zunyi, 563006 Guizhou People’s Republic of China; 2 Hubei Key Laboratory of Embryonic Stem Cell Research, Faculty of Basic Medical Sciences, Hubei University of Medicine, Shiyan, 442000 Hubei People’s Republic of China; 3 Nursing College, Hubei Province Chinese Medicine Hospital, Hubei University of Traditional Chinese Medicine, Wuhan, 430065 Hubei People’s Republic of China; 4 Department of Physiology, Faculty of Basic Medical Sciences, Hubei University of Medicine, Shiyan, 442000 Hubei People’s Republic of China; 5 Institute of Basic Medical Sciences, Institute of Biomedicine, Hubei University of Medicine, Hubei, 442000 People’s Republic of China

**Keywords:** Myocardial infarction, Vagal nerve stimulation, Metabolism, Arteriogenesis, SDF-1α

## Abstract

Vagal nerve stimulation (VNS) provides a novel therapeutic strategy for injured hearts by activating cholinergic anti-inflammatory pathways. However, little information is available on the metabolic pattern and arteriogenesis of VSMCs after MI. VNS has been shown to stimulate the expression of CPT1α, CPT1β, Glut1, Glut4 and SDF-1α in coronary VSMCs, decreasing the number of CD68-positive macrophages while increasing CD206-positive macrophages in the infarcted hearts, leading to a decrease in TNF-α and IL-1β accompanied by a reduced ratio of CD68- and CD206-positive cells, which were dramatically abolished by atropine and mecamylamine in vivo. Knockdown of SDF-1α substantially abrogated the effect of VNS on macrophagecell alteration and inflammatory factors in infarcted hearts. Mechanistically, ACh induced SDF-1α expression in VSMCs in a dose-dependent manner. Conversely, atropine, mecamylamine, and a PI3K/Akt inhibitor completely eliminated the effect of ACh on SDF-1α expression. Functionally, VNS promoted arteriogenesis and improved left ventricular performance, which could be abolished by Ad-shSDF-1α. Thus, VNS altered the VSMC metabolism pattern and arteriogenesis to repair the infarcted heart by inducing SDF-1α expression, which was associated with the m/nAChR-Akt signaling pathway.

**Supplementary Information:**

The online version contains supplementary material available at 10.1007/s10735-023-10171-4.

## Introduction

 Clinically, ischemic heart disease is often caused by vascular stenosis or obstruction caused by atherosclerotic lesions in coronary arteries, which are related to endothelial injury, proliferation and migration in vascular smooth muscle cells (VSMCs) and abnormal activation of macrophages (Li et al. 2004a). Myocardial infarction (MI), characterized by an increased ratio of sympathetic nerve to vagus nerve activity, is accompanied by an inflammatory response and macrophage infiltration, leading to myocardial injury (Lim et al. 2016). Basic and clinical studies show that vagal nerve stimulation (VNS) rebalances autonomic nerve activity in injured hearts, providing a novel therapeutic strategy for autonomic dysfunction through activating cholinergic anti-inflammatory pathways (Jones et al. 1980; Li et al. 2004a; Lim et al. 2016; Martelli et al. 2014; Shen et al. 2011). However, the anti-inflammatory roles of VNS-mediated VSMCs in infarcted hearts remain unclear.

Indeed, alterations in the differentiated state of VSMCs play a critical role in the pathogenesis of atherosclerosis and intimal hyperplasia (Chiong et al. 2013; Peiró et al. 2016; Wall et al. 2018). Increased glucose transporter 1 (Glut1) expression in VSMCs promoted VSMC phenotype switching, monocyte recruitment and atherosclerosis through increased glycolysis and the polyol pathway in vascular stenosis and metabolic disease models (Adhikari et al. 2011; Hall et al. 2001; Kaiser et al. 1993; Peiró et al. 2016; Vesely et al. 2009; Wall et al. 2018). However, little information is available on the possible relationship between VSMC metabolic patterns and angiogenesis after MI.

As a promising therapeutic target with functional performance associated with both ionotropic and metabotropic signaling, α7-nicotinic ACh receptor (α7-nAChR) activation can improve the progression of Parkinson’s disease and Alzheimer’s disease (Fontana et al. 2023), in addition to its diverse roles in cancer pathogenesis (Hajiasgharzadeh et al. 2019). Significantly, recent reports have shown that reduced levels of α7-nAChR expression hamper angiogenesis in ischemic or infarcted hearts (Li et al. 2004b, 2019; Pillai et al. 2012; Yu et al. 2013). Conversely, VNS restored and even enhanced the levels of α7-nAChR and m3-AChR expression in the injured heart (Zhao et al. 2013), while it increased the concentration of ACh in the ventricle of the heart (Akiyama et al. 2001; Cookeet al. 2008; Coote et al. 2013; Kakinuma et al. 2005). Unfortunately, its potential mechanism has not been fully explored. As a classical molecule of chemokines, stromal cell-derived factor-la (SDF-1 alpha) can activate and/or induce the migration of hematopoietic/endothelial progenitor cells and endothelial cells, in addition to most leukocytes (Ceradiniet al. 2004; De Falco et al. 2004; Greenbaum et al. 2013; MacArthur et al. 2013). However, whether SDF-1α is involved in VNS-mediated anti-inflammatory and VSMC metabolism is yet to be determined.

In the present study, we found that VNS decreased the inflammatory response while increasing Glut1 expression in VSMCs of the infarcted hearts. Therefore, we hypothesized that VNS optimized VSMC metabolism patterns and inflammatory responses, resulting in improved cardiac function in infarcted hearts.

## Materials and methods

### Animals

In accordance with the Guide for the Care and Use of Laboratory Animals published by China and US National Institutes of Health, adult male Sprague‒Dawley (SD) rats (250–300 g) were supplied by the Experimental Animal Center of the Hubei University of Medicine. All animal protocols were approved by the Institutional Animal Care and Use Committee of Hubei University of Medicine.

### Model establishment

The left anterior descending coronary artery (LAD) in rats was ligated to prepare the MI model as previously described (Tang et al. 2011). In brief, ketamine (50 mg/kg, i.p.) and xylazine (10 mg/kg, i.p.) were used to anesthetize D rats (250–300 g). LAD ligation was promptly performed after thoracotomy on the left fourth intercostal space after tracheal ventilation remained stable using a Columbus ventilator (HX-300, Taimeng Instruments, Chengdu, China). When confirming the occurrence of myocardial infarction with blanching of the myocardium as well as electrocardiography, the open thoracic cavity was sutured layer by layer immediately.

### Vagus nerve stimulation

Surviving rats were randomized into groups with sham or stimulation on the 7th day of myocardial infarction. After the left and right vagal nerves in the neck were gently exposed and separated, the vagal nerve was looped with Tefloncoated stainless-steel wires (UL1330; Triumph Cable Co, Ltd, China) and linked into the stimulator output part (BL-420 S; Chengdu Tme Technology Co, Ltd, China) for electrical stimulation (20 Hz for 10 s every minute for 4 h) as previously described (Luo et al. 2020). Regular pulse stimulation in the vagal nerve was executed in the vagus nerve stimulation group (VNS) (Luo et al. 2020). Similar operations were performed without initiating stimulation in the sham group (Sham) and MI group (MI). A 10% reduction in heart rate was used as a criterion for VNS. A mixture of white petrolatum (Vaseline) and paraffin was used to immerse the vagus nerve, and electrodes were connected to prevent drying. The inhibition of the inflammatory response after VNS was used as an endpoint criterion, marked by the number of decreased macrophages.

### ACh receptor inhibitor treatment in vivo

One hour before VNS, mancamylamine (MLA, 10 mg/kg, ip) or atropine (Atrop, 10 mg/kg, ip) was used to assess whether the role of VNS in SDF-1α expression in the infarcted heart was linked to mACh-R and α7-nAChR as described previously (Zhao et al. 2013; Luo et al. 2020). In brief, 7 days after MI, MI rats were randomly divided into four groups, namely, the MI group (MI), MI-VNS group (VNS), MI-VNS-MLA group (VNS + MLA), and MI-VNS-Atrop group (VNS + Atrop). One hour before VNS, the rats in the VNS + MLA and VNS + Atrop groups were injected with MLA (10 mg/kg, ip) or Atrop (10 mg/kg, ip), respectively. Meanwhile, the rats in the MI and VNS groups were injected with an equivalent solvent solution. Finally, the rats in the VNS, VNS + MLA and VNS + Atrop groups were treated by regular pulse stimulation of the vagal nerve. Meanwhile, similar operations were executed in the rats for the Sham and MI groups without initiating stimulation.

### Ad-shSDF-1α preparation and knockdown of SDF-1α injection in vivo

To determine the effects of VNS on VSMC metabolism and arteriogenesis/angiogenesis in infarcted hearts, an adenovirus carrying SDF-1α shRNA (Ad-shSDF-1) was used for local injection into the myocardium. Because Ad-shSDF-1 was injected locally into the myocardium, in addition to transfection into the VSMCs of hearts, Ad-shSDF-1 could also be transfected into other cells within the myocardium. In this experiment, the effects of knocking down SDF-1α could be related to both VSMCs and other cells. Ad-shCtrl and Ad-shSDF-1 were designed using a dedicated program provided by our published data (Tang et al. 2011). Ad-shCtrl and Ad-shSDF-1α (1 × 109 pfu in 200 µL) were injected into four sites of the infarcted hearts (50 µL per site, 12 rats/group) with a 30-gauge tuberculin syringe 3 days before VNS. Two injections were in the myocardium bordering the ischemic area, and two were within the ischemic area (Tang et al. 2011). Penicillin (150,000 U/mL, i.v.) was given before each procedure. Buprenorphine hydrochloride (0.05 mg/kg, s.c.) was administered twice a day for the first 48 h after the procedure.

### Measurement of hemodynamic parameters

Twenty-eight days after treatment, heart functions, including left ventricular systolic pressure (LVSP), left ventricular end-diastolic pressure (LVEDP), and rate of the rise and fall of ventricular pressure (+ dP/dt_max_ and – dP/dt_max_), were measured and evaluated using BL-420s (Chengdu Tai-meng, Co, China) as described previously (Tang et al. 2015). Following the anesthesia of rats with the application of ketamine (50 mg/kg, i.p.) and xylazine (10 mg/kg, i.p.), one end of the catheter was connected to a pressure transducer, and the other end of the catheter filled with heparinized (10 U/mL) saline solution was advanced into the left ventricle to record ventricular pressure while it was inserted into the isolated left carotid artery. After evaluating cardiac function, the heart was immediately collected for subsequent detection and analysis.

### ELISA for ACh detection

According to the manufacturer’s protocol (ab65345, Abcam), ELISA for ACh in heart tissues and blood samples treated with the cholinesterase inhibitor eserine (100 µM) was performed to confirm these changes in ACh levels in cardiac tissue and serum after VNS.

### Immunostaining

 Heart tissue serial transverse sections (5 μm) were prepared as previously described (Tang et al. 2011). Before adding primary antibodies, a blocking buffer (PBS containing 5% goat serum and 0.1% Triton X-100) was used to treat these sections at room temperature for 1 h. The primary antibodies (diluted in blocking buffer), including goat anti-rat SDF-1α (sc-6193, 1:150; Santa Cruz), mouse anti-rat α-SMA (SC-130,616, 1:150; Santa Cruz), m1-m5-AChR (1:150; Santa Cruz), mouse anti-rat CD31 (ab24590, 1:250; Abcam), CD206 (245,955, 1:200; CST) and rabbit anti-rat CD68 (1:250, GB11067, ServiceBio), were incubated at 4 °C overnight, and then the secondary antibodies, including horseradish peroxidase (HRP)-labeled goat anti-mouse IgG, goat-anti-rabbit IgG and rabbit-anti-goat IgG were incubated at room temperature for 2 h (Tang et al. 2011; Cao et al. 2017). Eventually, these indicated results were quantified by densitometry analysis (Image Pro, USA) after taking pictures under a microscope (MF43-N, Olympus, Japan) (Tang et al. 2018). The corresponding negative controls for the antibodies have been executed, showing no nonspecific binding.

### Cell cultures and groups

Human vascular smooth muscle cells (HVSMCs) (Jennio Biotech Co. Ltd, Guangzhou, China) were cultured in complete medium as previously mentioned (Lv et al. 2018). To further assess the mechanisms underlying ACh-induced SDF-1α expression in HVSMCs, the mACh-R blocker atropine (1 µM) or nACh-R blocker mecamylamine (7 µM), PI3K/Akt inhibitor wortmannin (50 nM), eNOS inhibitor L-NAME (300 µM), MEK/ERK1/2 inhibitor PD98059 (50 µM), and p38MAPK inhibitor SB203580 (30 µM) were inoculated for 1 h before treatment with 10^−5^ M ACh for 24 h. For subsequent target molecular detection, these harvested cells were lysed in RAPI buffer containing protease and phosphatase inhibitors (Luo et al. 2020).

### Western blot

Western blotting was carried out with primary antibodies against AKT (1:1000, Cell Signaling, #9272s), pAKT (1:1000, Cell Signaling, #9271 s), SDF-1α (sc-6193, 1:150, Santa Cruz), and α-tubulin (T9026, 1:5000, Sigma). The samples were collected and homogenized on ice in 0.1% Tween-20 homogenization buffer containing protease inhibitors. Fifty micrograms of protein was resolved on a 10% SDS‒PAGE gel and transferred onto a polyvinylidene fluoride (PVDF) membrane (Millipore). After being blocked with 5% nonfat milk, the membrane was incubated with primary antibody (1:1000 dilutions) for 90 min followed by incubation with horseradish peroxidase (HRP)-conjugated secondary antibodies (1:10000, Jackson ImmunoResearch). Protein expression was visualized by enhanced chemiluminescence and quantified by densitometry (Tang et al. 2011).

### Measurement of vascular density

The numbers of α-SMA or CD31-positive vessels in each section were analyzed in 6–8 equally distributed areas of 0.1 mm^2^ in the peri-infarction or infarction area. The values were then expressed as the number of vessels per squared millimeter. Two independent examiners analyzed these results using the Image-Pro Plus software package (Media Cybernetics, Carlsbad, CA) (Lv et al. 2018; Tang et al. 2011).

### Statistical analyses

IBM SPSS statistics software (version 22, 32-bit editing) was used for statistical analysis. https://www.ibm.com/cn-zh/products/spss-statistics.The data shown are the mean ± SD. Statistical significance between two groups was determined by paired or unpaired Student’s *t* test. The results for more than two experimental groups were evaluated by one-way ANOVA to specify differences between groups. *P <* 0.05 was considered significant.

## Results

### VNS increased CPT1α expression in VSMCs of the infarcted hearts

**Fig. 1:**
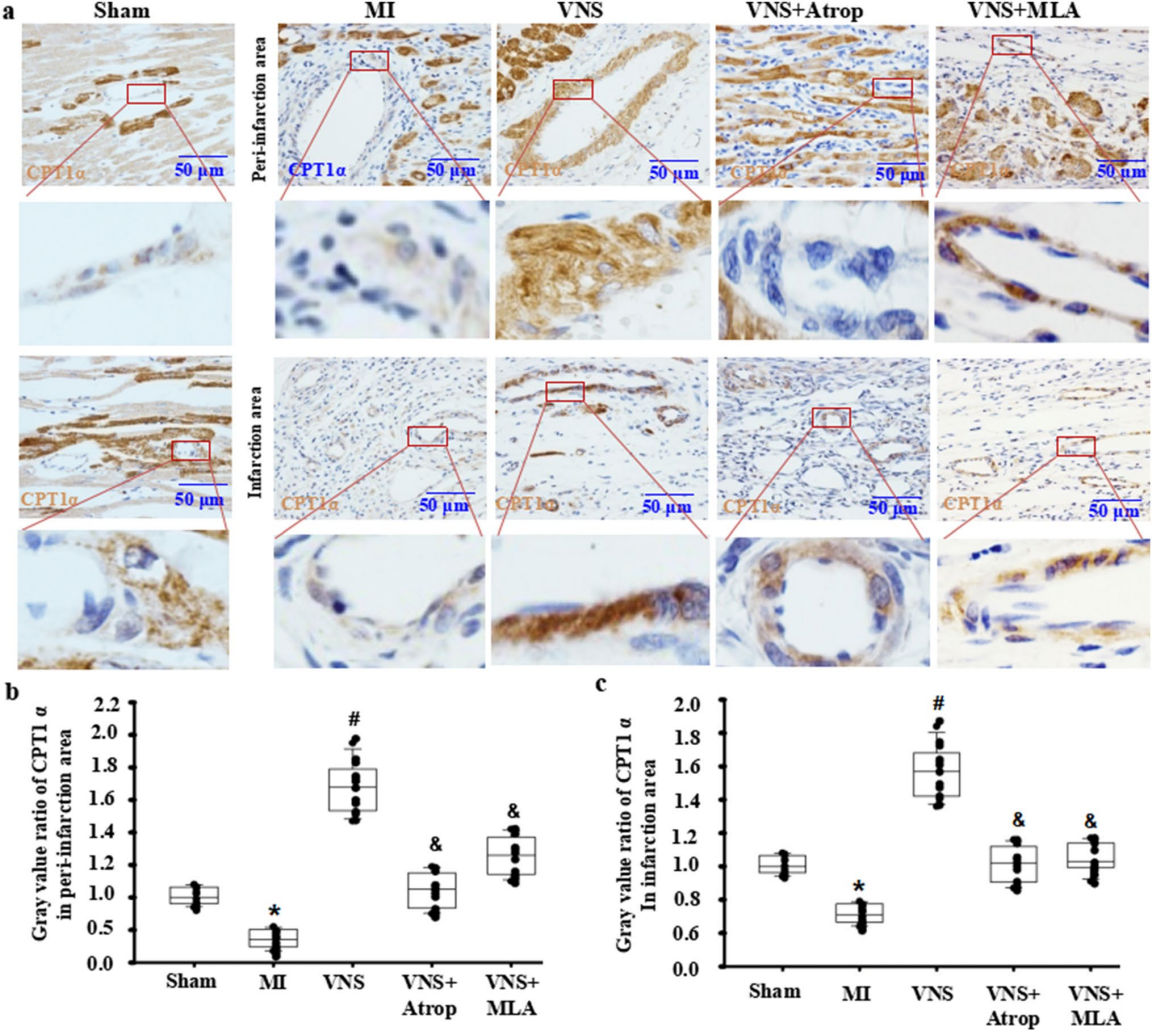
VNS increased CPT1α expression in VSMCs in infarcted hearts. **a** Typical immunostaining images for CPT1α showing that VSMCs were CPT1α positive, and VNS increased CPT1α expression in VSMCs of infarcted heart, which could be abolished by atropine (Atrop) or mecamylamine (MLA). Brownish yellow indicates CPT1α; hematoxylin-stained nucleus. **b**, **c** Semiquantitative analysis of CPT1α levels in VSMCs of the peri-infarction area (**b**) and infarction area (**c**) of the infarcted hearts. *P < 0.05 vs. Sham; # P < 0.05 vs. MI; & P < 0.05 vs. VNS (n = 12)

To observe the traits of CPT1α expression in the heart, using immunohistochemical staining, we first found that CPT1α expression in cardiomyocytes showed higher levels near the blood vessels in normal sham-operated hearts, while its levels were lower in blood vessels (Fig. 1A). After MI, CPT1α expression was not altered in cardiomyocytes in the peri-infarction area but was obviously decreased in cardiomyocytes in the infarction area (Fig. 1A–C). Then, VNS induced CPT1α expression in vessels of the peri-infarction and infarction areas in infarcted hearts, especially in VSMCs, in addition to cardiomyocytes (Fig. 1A–C). Last, to confirm the relationship between VNS’s effect and cholinergic receptors, we used the m-ACh-R inhibitor atropine (0.5 mg/kg, ip) or the α7-nACh-R blocker mecamylamine (1.0 mg/kg, ip) to treat the infarcted heart 1 h after MI and found that the increased CPT1α expression induced by VNS could be obviously abolished by atropine or mecamylamine, respectively. These results demonstrated that VNS induced CPT1α expression in the infarcted hearts, especially in the VSMCs of heart vessels, which was related to m/n-ACh-R.

### VNS increased CPT1β expression in VSMCs of the infarcted hearts

**Fig. 2:**
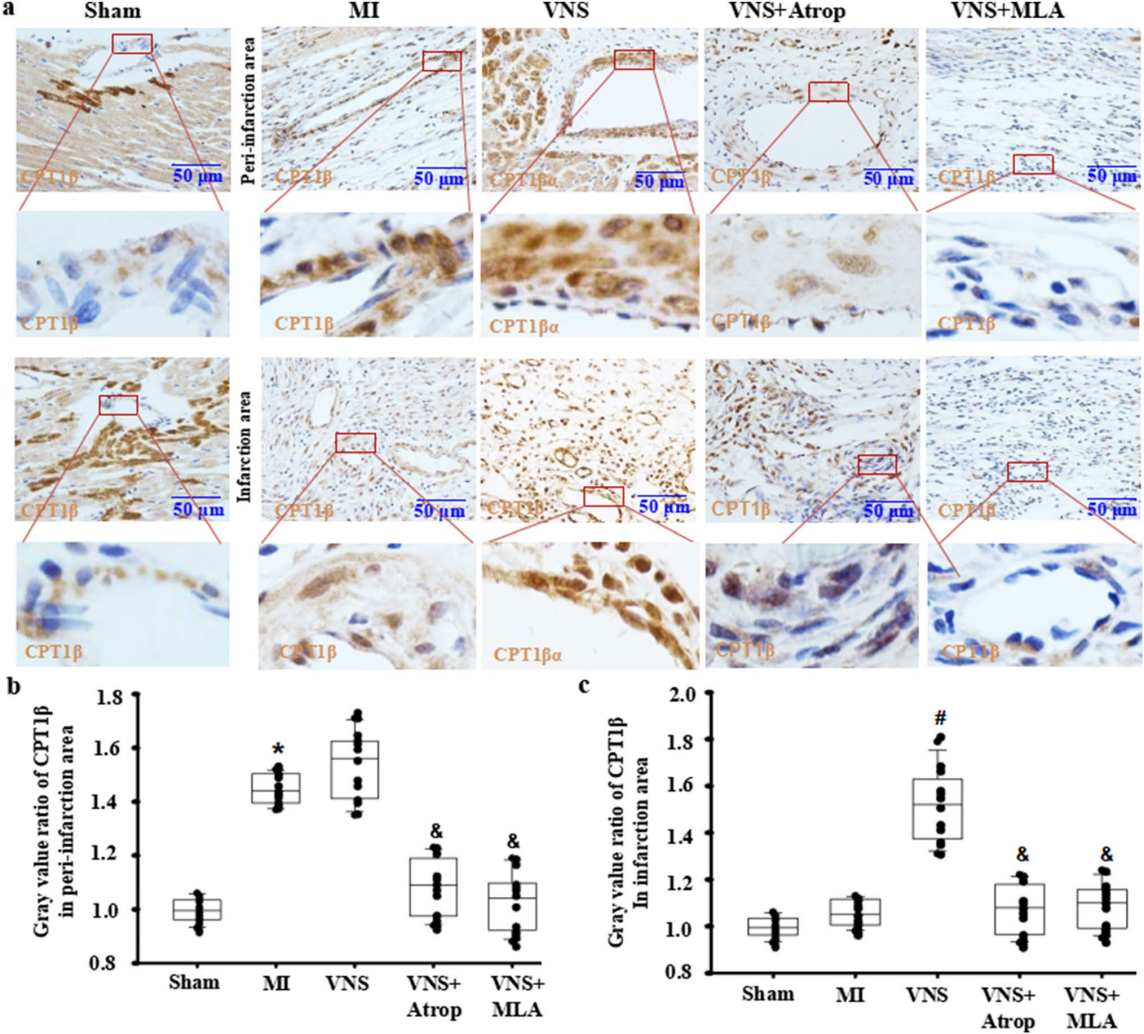
VNS increased CPT1β expression in VSMCs of the infarcted hearts. **a** Typical immunostaining images for CPT1β showing that VSMCs were CPT1β positive, and VNS increased CPT1β expression in VSMCs of infarcted heart, which could be abolished by atropine (Atrop) or mecamylamine (MLA). Brownish yellow indicates CPT1β; Hematoxylin-stained nucleus; **b**, **c** Semiquantitative analysis of CPT1β levels in VSMCs of the peri-infarction area (**b**) and infarction area (**c**) of the infarcted hearts. *P < 0.05 vs. Sham; # P < 0.05 vs. MI; & P < 0.05 vs. VNS (n = 12)

To reveal the characteristics of CPT1β expression in the heart, using immunohistochemical staining, we first found that CPT1β expression in cardiomyocytes was higher near blood vessels in sham-operated hearts but lower in blood vessels (Fig. 2A–C). After MI, CPT1β expression was decreased in cardiomyocytes in both the peri-infarction and infarction areas (Fig. 2A). Then, VNS induced CPT1β expression in vessels of the peri-infarction and infarction areas in infarcted hearts, especially in VSMCs of the infarction area, in addition to cardiomyocytes near the blood vessels (Fig. 2A–C). More importantly, we found that VNS-mediated CPT1β expression could be obviously abolished by atropine or mecamylamine, indicating that VNS induced CPT1β expression in the infarcted hearts, especially in the VSMCs of heart vessels.

### VNS slightly increased Glut1 expression in VSMCs of the infarcted hearts

**Fig. 3:**
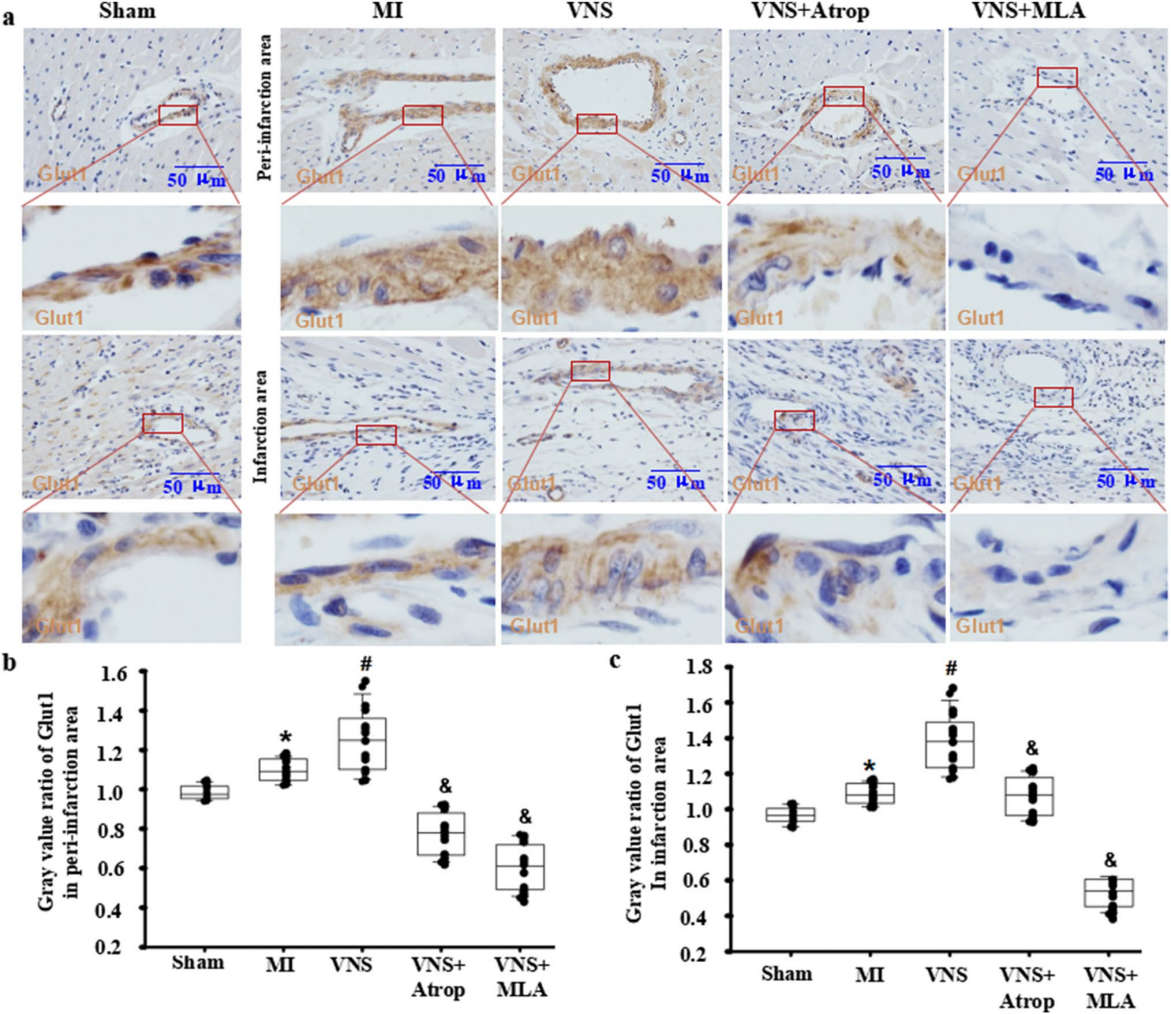
VNS slightly increased Glut1 expression in VSMCs of the infarcted hearts. **a** Typical immunostaining images for Glut1 showing that VSMCs were CPT1β positive, and VNS increased Glut1 expression in VSMCs of infarcted heart, which could be abolished by atropine (Atrop) or mecamylamine (MLA). Brownish yellow indicates Glut1; Hematoxylin-stained nucleus; **b**, **c** Semiquantitative analysis of Glut1 levels in VSMCs of the peri-infarction area (**b**) and infarction area (**c**) of the infarcted hearts. *P < 0.05 vs. Sham; # P < 0.05 vs. MI; & P < 0.05 vs. VNS (n = 12)

To confirm the traits of Glut1 expression in the heart, using immunohistochemical staining, we first found that Glut1 expression in the vessels of hearts was higher in the sham-operation group, while whether near or far from the blood vessel, its expression levels in cardiomyocytes were low (Fig. 3A). After MI, Glut1 expression was not altered in cardiomyocytes but was slightly increased in vessels of both the peri-infarction and infarction areas, especially in VSMCs (Fig. 3A). Then, VNS did not markedly change Glut1 in cardiomyocytes but induced Glut1 expression in vessels of the peri-infarction and infarction areas in infarcted hearts, especially in VSMCs (Fig. 3A–C). Meanwhile, we found that VNS-induced Glut1 expression could be obviously abolished by atropine or mecamylamine.

### VNS slightly changed Glut4 expression in VSMCs of the infarcted hearts

**Fig. 4:**
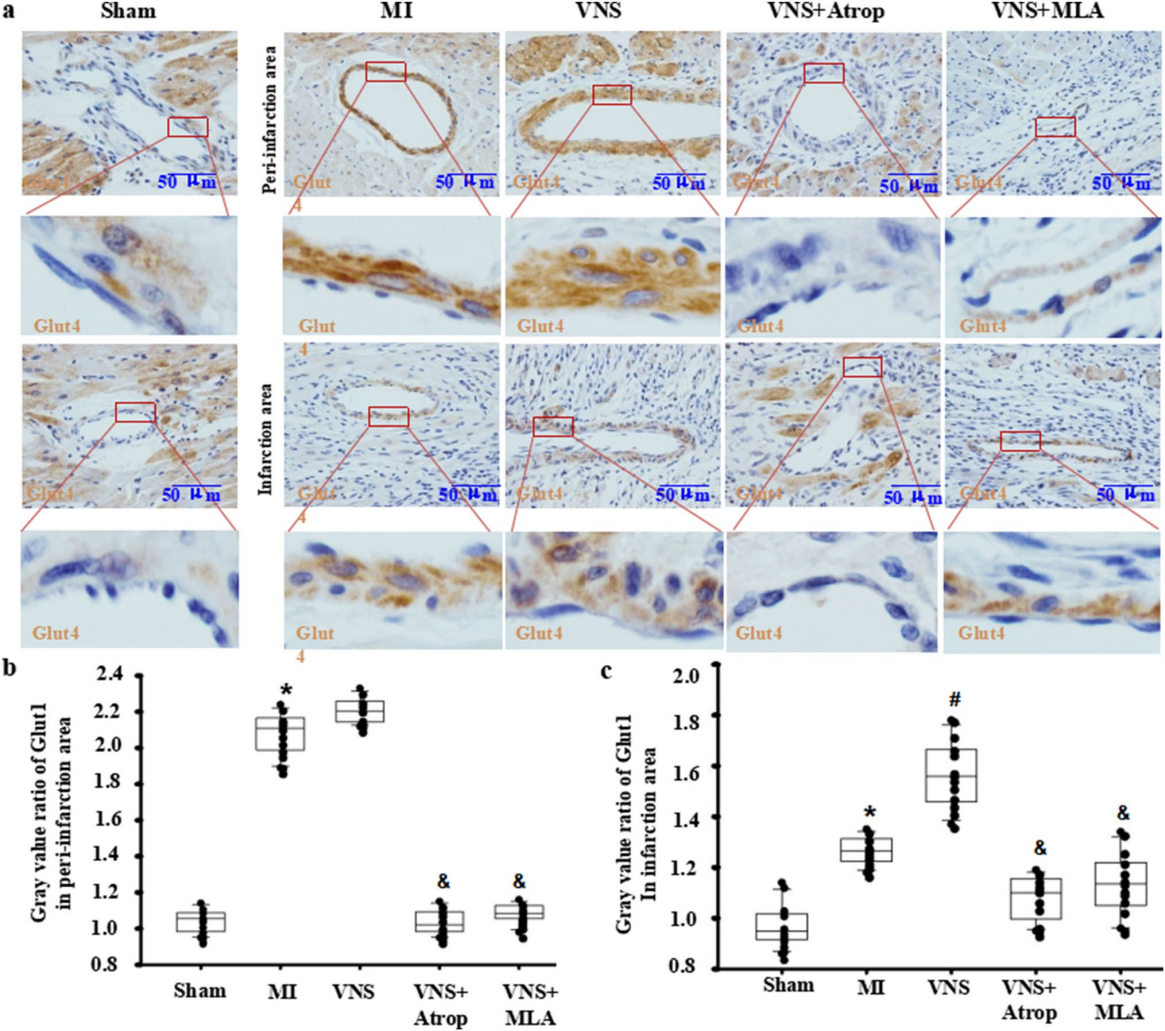
VNS slightly changed Glut4 expression in VSMCs of the infarcted hearts. **a**, **b** Typical immunostaining image of Glut4 in the infarction area and peri-infarction area of infarcted hearts treated with mecamylamine (MLA, 10 mg/kg, ip) and atropine (Atrop, 10 mg/kg, ip) before VNS. Brownish yellow indicates Glut4; hematoxylin-stained nucleus. **b**, **c** Semiquantitative analysis of Glut4 levels in VSMCs in the peri-infarction area (**b**) and infarction area (**c**) of infarcted hearts. *P < 0.05 vs. Sham; # P < 0.05 vs. MI; & P < 0.05 vs. VNS (n = 12)

To confirm the traits of Glut4 expression in the heart, using immunohistochemical staining, we first found that Glut4 expression in cardiomyocytes of hearts showed higher levels in the sham-operation group, while its expression levels in vessels were low (Fig. 4A). After MI, Glut4 expression was decreased in cardiomyocytes but increased in vessels in both the peri-infarction and infarction areas, especially in VSMCs (Fig. 4A). Then, VNS markedly increased Glut4 expression in cardiomyocytes in the peri-infarction area in the infarcted hearts but not in the peri-infarction area of the infarcted hearts. Furthermore, VNS obviously increased Glut4 expression in vessels of the infarction area in infarcted hearts, especially in VSMCs, but not in the peri-infarction of infarcted hearts (Fig. 4A–C). Last, we found that VNS-induced Glut4 expression could be obviously abolished by atropine or mecamylamine, indicating that VNS induced Glut4 expression in both cardiomyocytes and VSMCs of the infarcted hearts through m/n-AChR. These results showed that VNS induced Glut4 expression in the infarcted hearts, especially in VSMCs of heart vessels.

### VNS altered M1/M2 macrophages in myocardial infarction through m/n-AChR-SDF-1α

**Fig. 5:**
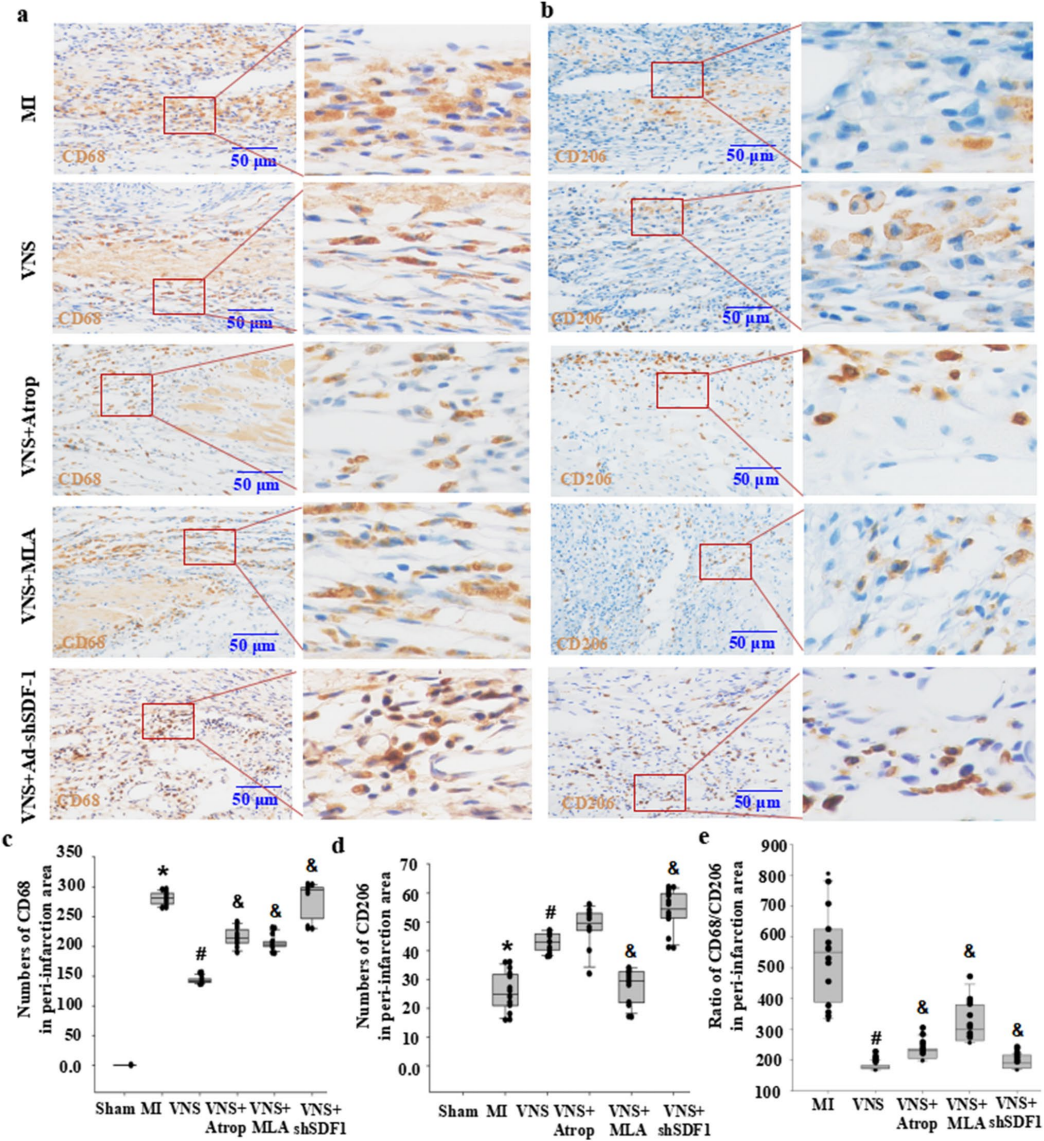
VNS altered M1/M2 macrophages in the peri-infarction area of myocardial infarction through m/n-AChR-SDF-1α. **a**, **b** Typical immunostaining image of CD68 (M1 macrophages) and CD206 (M2 macrophages) in the peri-infarction area of infarcted hearts treated with mancamylamine (MLA, 10 mg/kg, ip), atropine (Atrop, 10 mg/kg, ip) or SDF-1α knockdown by shRNA (Ad-shSDF-1) before VNS. Brownish yellow indicates CD68 or CD206; hematoxylin-stained nucleus. **c**, **d** Semiquantitative analysis of CD68 and CD206 numbers in the peri-infarct area of the infarcted hearts. *P < 0.05 vs. Sham; # P < 0.05 vs. MI; & P < 0.05 vs. VNS (n = 12). **e** Semiquantitative analysis of the ratio of CD68/CD206 levels in the peri-infarct area of the infarcted hearts. *P < 0.05 vs. Sham; # P < 0.05 vs. MI; & P < 0.05 vs. VNS (n = 12)

**Fig. 6:**
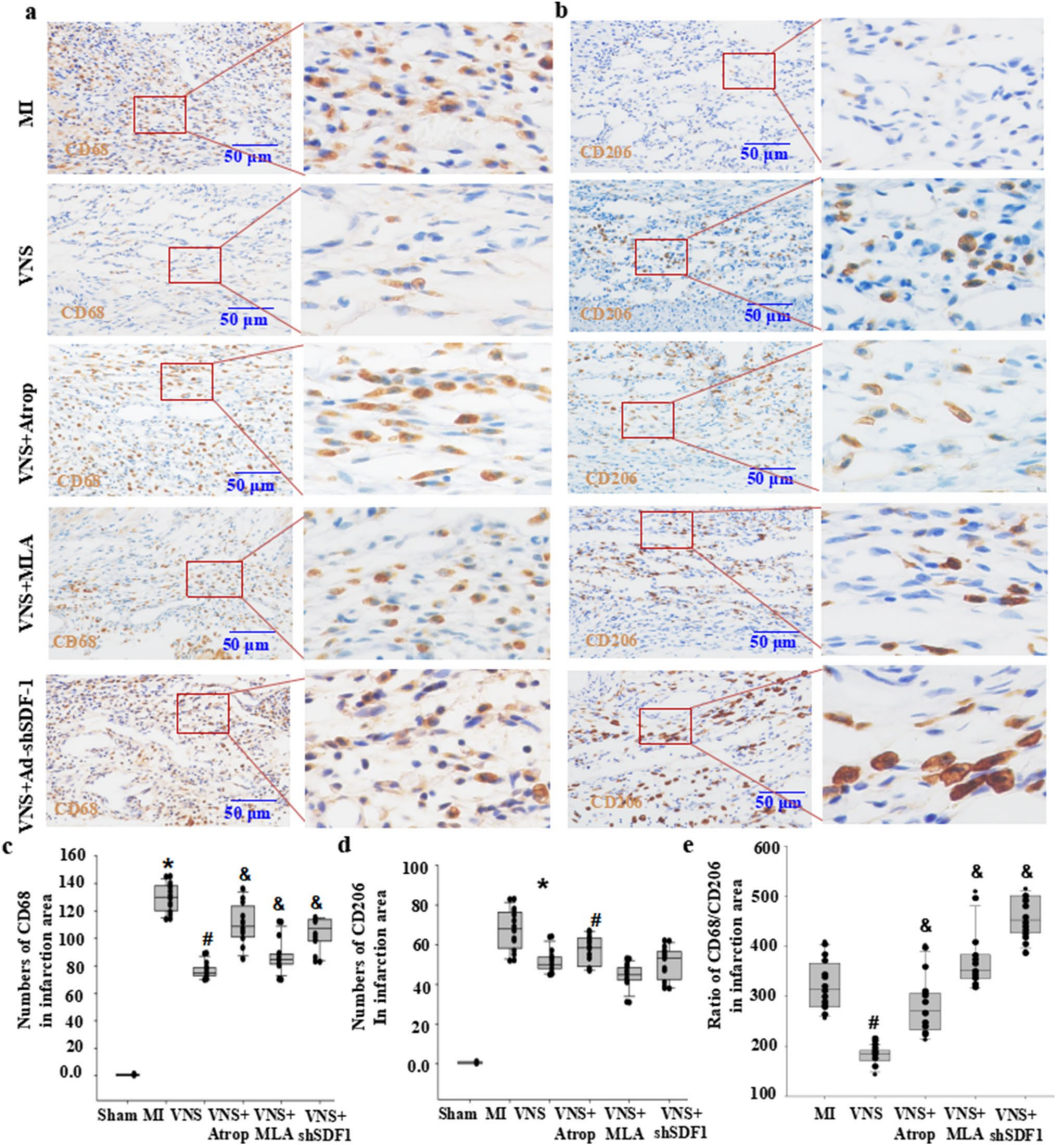
VNS altered M1/M2 macrophages in the infarction area of myocardial infarction through m/n-AChR-SDF-1α. **a**, **b** Typical immunostaining image of CD68 (M1 macrophages) and CD206 (M2 macrophages) in the infarction area of infarcted hearts treated with mancamylamine (MLA, 10 mg/kg, ip), atropine (Atrop, 10 mg/kg, ip) or SDF-1α knockdown by shRNA (Ad-shSDF-1) before VNS. Brownish yellow indicates CD68 or CD206; hematoxylin-stained nucleus. **c**, **d** Semiquantitative analysis of CD68 levels in the infarction area of the infarcted hearts. *P < 0.05 vs. Sham; # P < 0.05 vs. MI; & P < 0.05 vs. VNS (n = 12). **e** Semiquantitative analysis of the ratio of CD68/CD206 levels in the infarction area of the infarcted hearts. *P < 0.05 vs. Sham; # P < 0.05 vs. MI; & P < 0.05 vs. VNS (n = 12)

To confirm whether VNS is involved in macrophages in infarcted hearts, we used immunohistochemical staining for the M1 macrophage marker CD68 and the M2 macrophage marker CD206, as shown in Figs. 5A and E and 6A and E. Compared with the sham group, the numbers of CD68-positive macrophages were increased, while CD206-positive macrophagecell numbers were decreased, resulting in increased levels of TNFα and IL-1β (Supplemental Fig. S2) in the infarcted hearts accompanied by an increased ratio of CD68 and CD206 (Figs. 5 and 6). Instead, VNS obviously decreased the number of CD68-positive cells in infarcted hearts. Although CD206 macrophagecell numbers in VNS-treated MI hearts did not obviously increase compared with MI hearts, the ratio of CD68 and CD206 was markedly reduced in the infarcted hearts, leading to decreased levels of TNFα and IL-1β (Supplemental Fig. S2). Both atropine and mecamylamine abolished the effects of VNS on macrophages and inflammatory factors in infarcted hearts (Figs. 5 and 6). Meanwhile, VNS increased the expression of SDF-1α in VSMCs of infarcted hearts, and local injection of Ad-shSDF-1α into the infarcted hearts obviously abolished the induced SDF-1α expression in infarcted hearts by VNS (Supplemental Fig. S1). Furthermore, knockdown of SDF-1α in the infarcted hearts obviously abrogated the effect of VNS on macrophages and inflammatory factors in the infarcted hearts (Figs. 5 and 6). These results indicated that VNS altered the ratio of M1/M2 macrophages in myocardial infarction through SDF-1α.

### VNS promoted arteriogenesis and its matching CPT1α/β and Glut1/4 expression in the infarcted hearts involved in SDF-1α

**Fig. 7:**
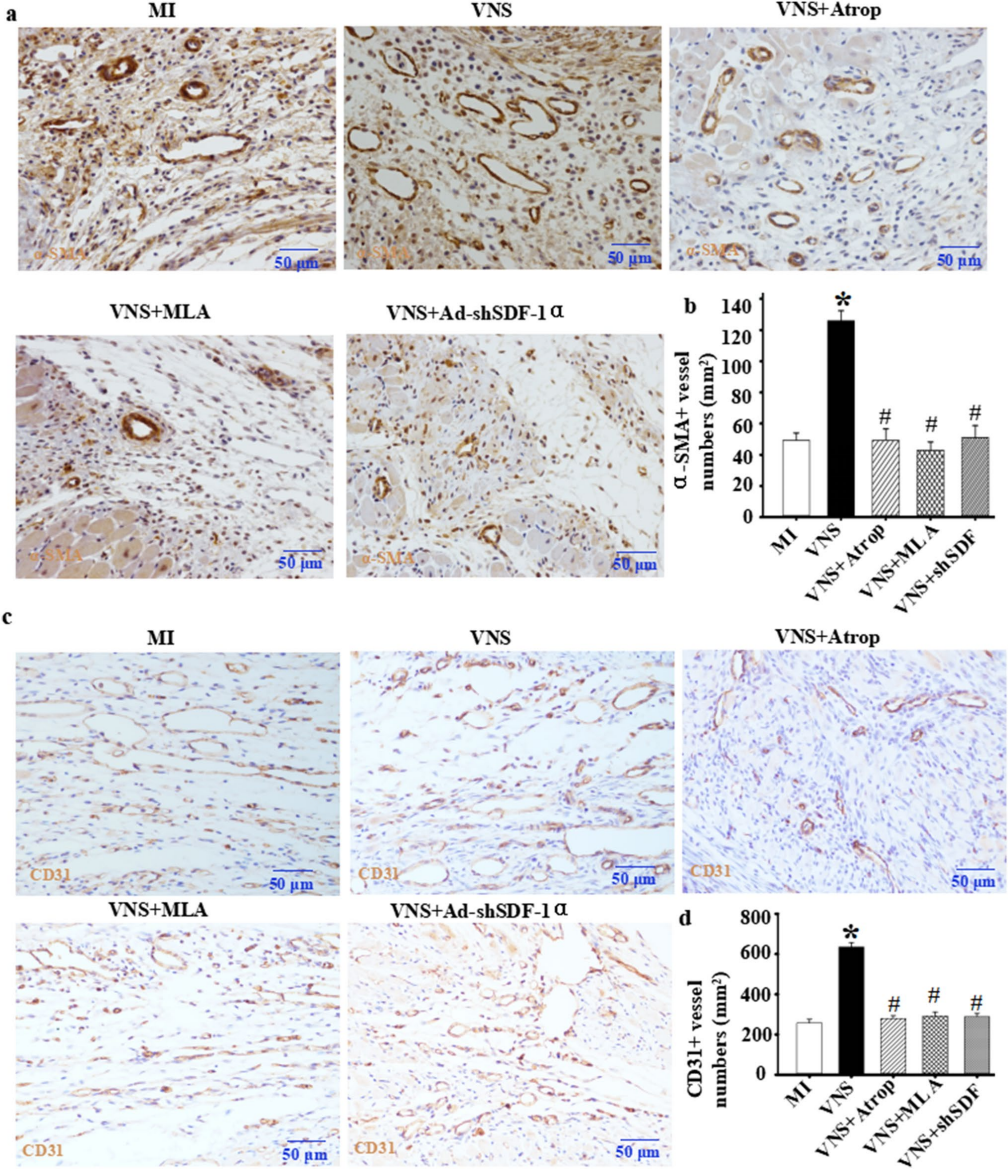
VNS increased the number of vessels in infarcted hearts. **a** Typical image of α-SMA-positive vessels in infarcted hearts as determined by immunostaining of α-SMA. **b** Quantitative analysis of α-SMA-positive vessels in infarcted hearts. *P < 0.05 vs. MI; # P < 0.05 vs. VNS. (n = 6). **c** Typical image of CD31-positive vessels in infarcted hearts as determined by immunostaining of CD31. **d** Quantitative analysis of CD31-positive vessels in infarcted hearts. *P < 0.05 vs. MI; # P < 0.05 vs. VNS. (n = 6)

**Fig. 8:**
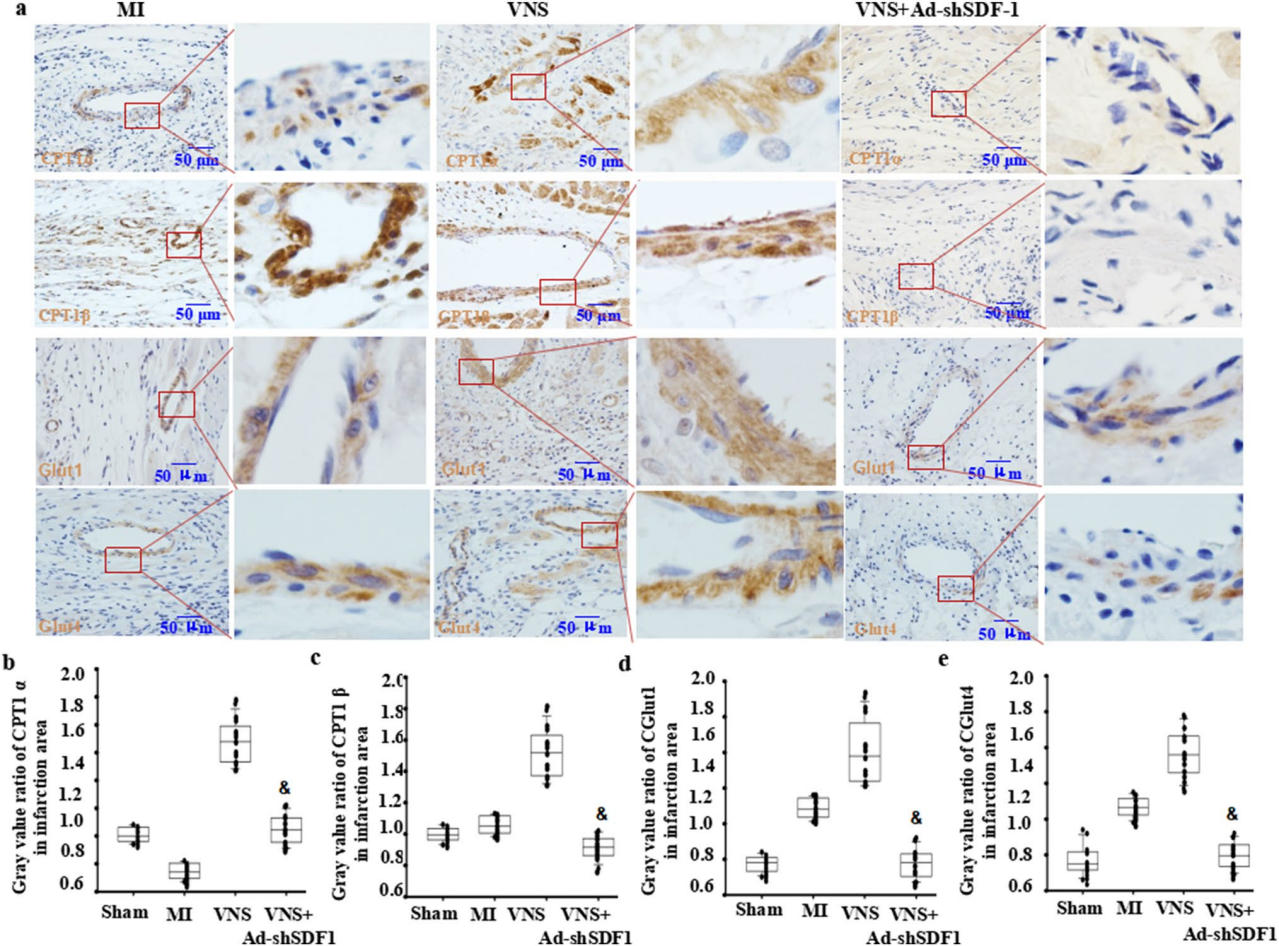
VNS-mediated CPT1α/β and Glut1/4 expression in the infarcted heart involves SDF-1α. **a** Typical immunostaining image of CPT1α/β and Glut1/4 in the infarction area and peri-infarction area of infarcted heart treated with knockdown of SDF-1α by shRNA (Ad-shSDF-1) before VNS. Brownish yellow indicates CPT1α/β or Glut1/4; hematoxylin-stained nucleus. **b**, **c** Semiquantitative analysis of CPT1α/β (**b**) and Glut1/4 (**c**) levels in the infarcted hearts. *P < 0.05 vs. Sham; # P < 0.05 vs. MI; & P < 0.05 vs. VNS (n = 12)

To explore whether VNS-induced SDF-1α is involved in arteriogenesis in infarcted hearts, we used immunohistochemical staining for α-SMA, as shown in Fig. 7A and B. VNS increased the number of α-SMA-positive vessels in infarcted hearts, and the specific effects could be abolished by mAChR or nAChR blockade or SDF-1α knockdown by shRNA. Then, to confirm whether VNS-induced SDF-1α was involved in CPT1α/β and Glut1/4 expression in the vessels of the infarcted hearts, we used immunohistochemical staining for CPT1α/β and Glut1/4, and similar to the previous results showing that VNS altered CPT1α/β and Glut1/4 expression in the infarcted hearts (Figs. 1, 2, 3 and 4), local injection of Ad-shSDF-1α into the infarcted hearts obviously abolished the induction of CPT1α/β and Glut1/4 in the infarcted hearts by VNS, indicating that VNS-induced CPT1α/β and Glut1/4 expression in the infarcted hearts could be related to VNS-induced SDF-1α expression (Fig. 8).

### ACh induced SDF-1α in VSMCs through the m/n-AChR-Akt signaling pathway

**Fig. 9:**
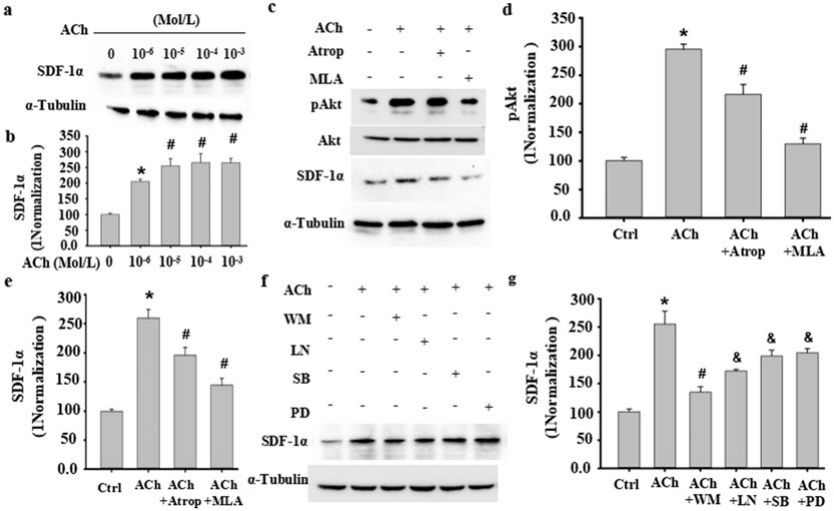
ACh induced SDF-1α in VSMCs through the m/n-AChR-Akt signaling pathway. **a**, **b** ACh dose-dependently induced SDF-1α expression in VSMCs, as detected by Western blot (**a**). (**b**) Semiquantitative analysis of SDF-1α expression in VSMCs. *P < 0.05 vs. 0 Mol/L ACh; #P < 0.05 vs. 10^−6^ Mol/L ACh (n = 3). **c**–**e** The increased SDF-1α and phosphorylation of Akt following ACh stimulation in VSMCs was abolished by the mACh-R inhibitor atropine (Atrop) or the nACh-R inhibitor mecamylamine, as determined by Western blot (**c**). **d**–**e** Semiquantitative analysis of SDF-1α expression in VSMCs. *P < 0.05 vs. 0 Mol/L ACh; # P < 0.05 vs. 10^−5^ Mol/L ACh (n = 3). **f**–**g** Signaling pathways mediating ACh-induced SDF-1α expression were assessed by pathway-specific inhibitors as indicated. Western blotting was used to detect ACh-induced SDF-1α expression in VSMCs following treatment with wortmannin (WM, 50 nM), LN (15 nM), SB203580 (SB, 30 µM), and PD98059 (PD, 50 µM). α-Tubulin served as an internal control. **g** Semiquantitative analysis of SDF-1α in Fig. 7f as indicated. At least three independent experiments were carried out. *P < 0.05 vs. 0 ACh (Ctrl); # P < 0.05 vs. 10^−5^ Mol/L ACh; # P < 0.05 vs. ACh + WM (n = 3)

To confirm the relationship between ACh and SDF-1α mediated by VNS, in vitro VSMCs were treated with different dosages of ACh, as shown in Fig. 9A and B. ACh induced SDF-1α expression in a concentration-dependent manner. ACh-induced SDF-1α was evidently abrogated by atropine and mecamylamine (Fig. 9C and E). Furthermore, ACh-induced SDF-1α could be dramatically abolished by the PI3K/Akt inhibitor wortmannin (WM) (Fig. 9F and G). These results indicated that ACh could induce SDF-1α in VSMCs through the m/n-AChR-Akt signaling pathway.

### VNS improved cardiac function

**Fig. 10:**
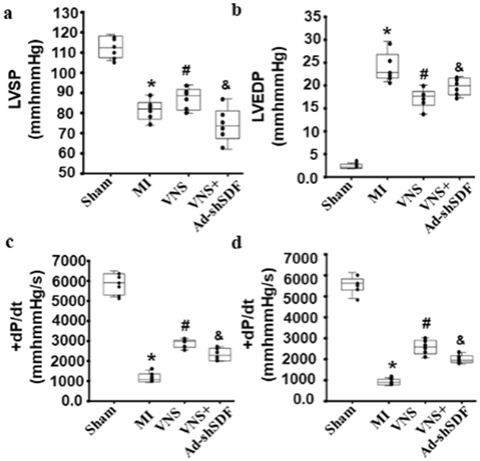
VNS improved cardiac function. **a**–**d** Left ventricular systolic pressure (LVSP, a), left ventricular end-diastolic pressure (LVEDP, b), rate of rise of ventricular pressure (+ dP/dt max , c), and rate of fall of ventricular pressure (– dP/dt max , d) in the VNS group were obviously improved, which could be abolished by Ad-shSDF-1α. *P < 0.05 vs. Sham; # P < 0.05 vs. MI; & P < 0.05 vs. VNS. (n = 6)

Functional analysis was performed to explore whether VNS-induced angiogenesis improved infarcted heart function. The results showed that LV function, including LVSP, LVEDP, + dP/dt_max,_ and – dP/dt_max_, was significantly improved in VNS-treated hearts compared to MI-treated hearts. SDF-1α shRNA, however, abolished the VNS-improved LV function (Fig. 10).

## Discussion

Preclinical studies demonstrated that VNS could exert protective effects on the heart in animal models of myocardial ischemia‒reperfusion, MI and heart failure (Li et al. 2004a; Lim et al. 2016; Martelli et al. 2014; Shen et al. 2011). Multiple mechanisms involved in the beneficial effects of VNS on the heart. Typically, VNS not only improved parasympathetic tone and reflexes but also inhibited stellate ganglion nerve activity, leading to inhibition of proinflammatory cytokines. Recent findings have shown that VNS can reduce apoptosis and autophagy, suppress oxidative stress and optimize cardiac electrical stability and energy metabolism (Luo et al. 2020). In the present study, VNS increased the greater expression of CPT1α/β in VSMCs of the infarcted heart than Glut1/4 expression. VNS-induced SDF-1α expression decreased CD68-positive macrophagecell numbers while increasing CD206-positive macrophagecell numbers, leading to increased α-SMA-positive vessels and functional improvement in infarcted hearts. These results indicated that the protective effects of VNS could be involved in the optimization of VSMC metabolism patterns and arteriogenesis in infarcted hearts.

The cervical vagal nerve contains both afferent and efferent fibers composed of A-, B- and C-fibers (Bonaz et al. 2018). Theoretically, afferent fiber stimulation can be more beneficial for reducing sympathetic activity than efferent fiber stimulation. Regarding the frequency of stimulation, low frequencies (5–10 Hz) activate vagal afferents, whereas high frequencies (10–30 Hz) activate both vagal afferents and efferents (Castoro et al. 2011). Accumulated data have shown that low-level VNS exacts paradoxical effects from high-level VNS (Wang et al. 2019; Johnson et al. 2018). Indeed, in addition to alleviating inflammation, high-level VNS reduced the lower heart rate and inhibited cardiac remodeling (Wu et al. 2023). The reason may be the levels of autonomic imbalance, cardiac inflammation and heart failure, apart from lifestyle factors such as exercise. For example, VNS did not contribute to obvious improvement of heart function in patients with low levels of autonomic imbalance or long-standing heart failure (Sant’Anna et al. 2021). Conversely, VNS was dramatically beneficial for improving heart function in patients with cardiac inflammation (Johnson et al. 2018). In the present study, a frequency of 20 Hz was used, and we found that VNS markedly decreased inflammatory responses in the infarcted heart. These results indicated that the protective effects of VNS could be involved in the inflammatory modulation of the infarcted heart.

Acting as a typical stem cell chemotactic factor, SDF-1α expression could be controlled by inflammatory cytokines such as IL-1β and TNF-α. In the infarcted heart, the levels of IL-1β and TNF-α were obviously increased. VNS significantly reduced their levels in the infarcted heart, leading to increased expression of SDF-1α, in line with the evidence that IL-1β promoted SDF-1α expression, while TNF-α reduced its expression in various cells (Blaine et al. 2011; Krieger et al. 2016; Salvucci et al. 2004). At the same time, it could also align with a recent report that SDF-1α acted as an anti-inflammatory chemokine during autoimmune inflammatory responses (Mousavi et al. 2020). In addition, we found that ACh induced SDF-1α expression in VSMCs under normal culture conditions, which was related to the P13K/Akt signaling pathway. In summary, these results indicated that VNS at least in part promoted SDF-1α expression by regulating the inflammatory response.

Previous studies have shown that overexpression of Glut1 in VSMCs promotes chemokine CCL2 and monocyte chemoattractant protein-1 (MCP-1) expression and monocyte recruitment, resulting in excessive inflammatory reactions and accelerating atherosclerosis (Adhikari et al. 2011; Hall et al. 2001; Kaiser et al. 1993; Peiró et al. 2016; Vesely et al. 2009; Wall et al. 2018). Fortunately, we found that VNS did not induce Glut1 expression in the infarcted heart but obviously increased CPT1α/β expression and anti-inflammatory effects in the infarcted heart, in line with recent reports showing that increased CPT1α inhibited macrophage influx and the proinflammatory response to delay kidney fibrosis (Yuan et al. 2021). Unfortunately, the present study did not provide detailed information on how VNS regulates the molecular mechanism of CPT1α/β expression. We only showed superficial results of reduced CPT1α/β expression after treatment with atropine, mecamylamine or Ad-shSDF, considering the expression of mAChR and α7-nACh-R in VSMCs (Supplemental Figure S3). However, further in-depth research is needed to determine whether CPT1α/β, like Glut1, plays a role by regulating SDF-1α, as well as deeper mechanisms of CPT1α/β expression mediated by VNS and cholinergic receptors.

Of note, excessive VSMC proliferation and migration could cause atherosclerosis and vessel stenosis, resulting in various vessel-related diseases, including MI (Li et al. 2004b, 2019; Padro et al. 2020; Pestana et al. 2005). Both VSMC proliferation and migration and endothelialization are necessary for arteriogenesis and angiogenesis (Luo et al. 2020; Lv et al. 2018). Previous studies have shown that increased CPT1α contributes to VSMC proliferation, migration and survival, while excessive glucose metabolism damages VSMCs (Adhikari et al. 2011; Hall et al. 2001; Kaiser et al. 1993; Peiró et al. 2016; Vesely et al. 2009; Wall et al. 2018). In the present study, VNS obviously increased the expression of SDF-1α and CPT1α/β in VSMCs in infarcted hearts, leading to more α-SMA-positive vessels, which was consistent with the effects of SDF-1α and CPT1α/β on coronary sprouting and lymphangiogenesis (Das et al. 2019; Marín-Juez et al. 2019; Schoors et al. 2015; Wong et al. 2017). These results indicated that the optimized VSMC metabolism pattern induced by VNS could be involved in arteriogenesis and angiogenesis. The detailed mechanism of VNS involving SDF-1α and CPT1α/β during the process of arteriogenesis and angiogenesis needs further exploration in the future.

### Limitations

Due to the use of male rats in this study, there was no matching experiment with female rats. Previous studies have shown that VNS has more short-term effects in male rats than in female rats (Yaghouby et al. 2020; Yokota et al. 2022). Meanwhile, apart from electrode design and stimulated-side selection, the “dose” of electrical therapies, including current intensity, frequency and duty cycle, could affect the therapeutic effect of VNS on infarcted heart (De Ferrari et al. 2014; Jiao et al. 2016; Kong et al. 2012; Libbus et al. 2017; Nishizaki et al. 2016; Rousselet et al. 2014; Soltani et al. 2023; Wang et al. 2019; Wu et al. 2023; Yu et al. 2016). The present study mainly focused on the effects of VNS on the metabolic patterns of VSMCs and angiogenesis after MI. The different roles of different stimulating parameters in infarcted hearts should be explored in the future.

**Fig. 11:**
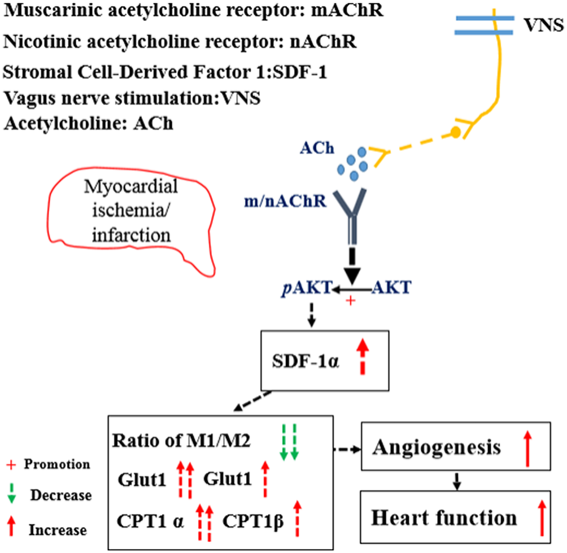
Working model. VNS improves VSMC metabolism and arteriogenesis in the infarcted heart through m/n-AChR-Akt1-SDF-1α. VNS and acetylcholine (ACh)-induced SDF-1α expression optimized the VSMC metabolism pattern characterized by increased CPT1α/β and Glut1/4 expression while promoting arteriogenesis in the infarcted heart, which was related to the ACh/m/nAChR/Akt signaling cascade

Taken together, our studies demonstrated that VNS improves heart function by optimizing VSMC metabolism patterns and decreasing inflammatory responses in the infarcted heart via activation of SDF-1α signaling (Fig. 11).

### Supplementary Information

Below is the link to the electronic supplementary material.

Supplementary material 1 (DOCX 765.6 kb)

Supplementary material 2 (DOCX 2000.6 kb)

## Data Availability

Please contact the corresponding author for data requests.

## Abbreviations

ACh: Acetylcholine
α-SMA: Alpha smooth muscle actin
DMEM: Dulbecco’s modified Eagle’s medium
LVSP: Left ventricular systolic pressure
LVEDP: Left ventricular end-diastolic pressure
+dP/dt_max_: Rate of rise of ventricular pressure
–dP/dt_max_: Rate of fall of ventricular pressure
M: Mol/L
SDF-1α: Stromal cell derived factor 1 alph
