# Supplementary material for: VNS improves VSMC metabolism and arteriogenesis in infarcted hearts through m/n-AChR-Akt-SDF-1α in adult male rats
Source: J Mol Histol. 2024 Jan 2;55(1):51–67. doi: 10.1007/s10735-023-10171-4 (PMC10830782; doi:10.1007/s10735-023-10171-4)
Supplement: Supplementary file 1 — Supplementary material 1 (DOCX 765.6 kb) [file 10735_2023_10171_MOESM1_ESM.docx]

**Supplemental files**

**VNS improves VSMC metabolism and arteriogenesis in infarcted hearts through m/n-AChR-Akt-SDF-1α in adult male rats**

Xing-yuan Li^1,2^*, Jia-Qi Liu^3^*, Yan Wang*, Yan Chen^1^, Wen-hui Hu^1^, Yan-xia Lv^2,4^, Yan Wu ^2,4^, Jing Lv^5#^, Jun-mingTang^2,4,5#^, De-ying Kong^1#^

^1^Department of Physiology, Faculty of Basic Medical Sciences, Zunyi Medicical University, Zunyi, Guizhou 563006, PR China

^2^Hubei Key Laboratory of Embryonic Stem Cell Research, Faculty of Basic Medical Sciences, Hubei University of Medicine, Shiyan, Hubei 442000, PR China

^3^Department of Physiology, Faculty of Basic Medical Sciences, Hubei University of Medicine, Shiyan, Hubei 442000, PR China

^4^Nursing College, Hubei University of Traditional Chinese Medicine, Wuhan, Hubei 430065, PR China

^5^Institute of Basic Medical Sciences, Institute of Biomedicine, Hubei University of Medicine, Hubei 442000, PR China

Xing-Yuan Li, E-mail: [836595262@qq.com](mailto:836595262@qq.com)

Jia-qi Liu, E-mail:1745456360@qq.com

Yan Wang, E-mail: 951331390@qq.com

Yan Chen, E-mail: 297379873@qq.com

Wen-hui Hu , E-mail: hwh199707@163.com

Yan-xia Lv, E-mail:350182495@qq.com

Yan Wu, E-mail: [2668224536@qq.com](mailto:2668224536@qq.com)

Jing Lv, E-mail: [389514970@qq.com](mailto:389514970@qq.com)

Jun-Ming Tang, E-mail: [tangjm416@163.com](mailto:tangjm416@163.com)

De-ying Kong, E-Mail:[vsysongyer@126.com](mailto:vsysongyer@126.com)

*Co-fisrt author : Xing-Yuan Li, Jia-qi Liu & Yan Wang

#Co-corresponding author: De-ying Kong, Jing Lv & Jun-Ming Tang, MD, PhD

Tel.:+86-719/8875312

E-mail: [vsysongyer@126.com](mailto:vsysongyer@126.com); tangjm416@163.com; [389514970@qq.com](mailto:389514970@qq.com)

**Supplemental. Method. S1. Ad-shSDF-1α prepare and Knockdown of SDF-1α *in vivo***

SDF-1α shRNA (shSDF-1) was designed using a dedicated program provided by our published data (Tang et al., 2011).To determine the effect of SDF-1α on angiogenesis in the infarcted heart, Ad-shCtrl, Ad-shSDF-1α (1×10^9^pfu in 200 μl) were injected into four sites of the infracted hearts (50 μl per site, 12 rats/group) with a 30-gauge tuberculin syringe 3 days before the VNS. Two injections were in the myocardium bordering the ischemic area and two within the ischemic area (Tang et al., 2011). Penicillin (150,000 U/mL, i.v.) was given before each procedure. Buprenorphine hydrochloride (0.05 mg/kg, s.c.) was administered twice a day for the first 48 hours after the procedure.

**Supplemental. Method. S2. Immunostaining**

Heart tissue serial transverse sections (5 μm) were prepared as mentioned (Tang et al., 2011). Before adding primary antibodies, a blocking buffer (PBS containing 5% goat serum and 0.1%Triton X-100) was used to treat these sections at room temperature for 1 hr. The primary antibodies (diluted in blocking buffer) including goat anti-rat SDF-1α (sc-6193,1:150; SantaCruz), mouse anti-rat α-SMA (SC-130616, 1:150; SantaCruz), and m1-m5-AChR (1:150; SantaCruz) were incubated at 4°C overnight; and then the secondary antibodies including horseradish peroxidase (HRP)-labeled goat anti-mouse IgG, goat-anti-rabbit IgG, FITC-conjugated anti-rabbit IgG, or TRITC-conjugated anti-mouse IgG (Jackson ImmunoResearch) were incubated at room temperature for 2 h, respectively (Tang et al., 2011; Cao et al., 2017). Eventually, these indicated results were quantified by analyzing densitometry (Image Pro, USA) after taking pictures under the microscope (MF43-N, Olympus, Japan) (Tang et al., 2018).

**Reference**

Cao, T., Zhang, L., Yao, L.-L., Zheng, F., Wang, L., Yang, J.-Y., et al. (2017). S100B promotes injury-induced vascular remodeling through modulating smooth muscle phenotype. Biochimica Et Biophysica Acta-Molecular Basis of Disease 1863(11), 2772-2782. doi: 10.1016/j.bbadis.2017.07.002.

Luo, B., Wu, Y., Liu, S.-l., Li, X.-y., Zhu, H.-r., Zhang, L., et al. (2020). Vagus nerve stimulation optimized cardiomyocyte phenotype, sarcomere organization and energy metabolism in infracted heart through FoxO3A-VEGF signaling. Cell Death & Disease 11(11). doi: 10.1038/s41419-020-03142-0.

Tang, J.-M., Luo, B., Xiao, J.-h., Lv, Y.-x., Li, X.-l., Zhao, J.-h., et al. (2015). VEGF-A promotes cardiac stem cell engraftment and myocardial repair in the infracted heart. International Journal of Cardiology 183, 221-231. doi: 10.1016/j.ijcard.2015.01.050.

Tang, J.M., Shi, N., Dong, K., Brown, S.A., Coleman, A.E., Boegehold, M.A., et al. (2018). Response Gene to Complement 32 Maintains Blood Pressure Homeostasis by Regulating α-Adrenergic Receptor Expression. Circ Res 123(9), 1080-1090. doi: 10.1161/circresaha.118.313266.

Tang, J.M., Wang, J.N., Zhang, L., Zheng, F., Yang, J.Y., Kong, X., et al. (2011). VEGF/SDF-1 promotes cardiac stem cell mobilization and myocardial repair in the infracted heart. Cardiovasc Res 91(3), 402-411. doi: 10.1093/cvr/cvr053.

Zhao, M., He, X., Bi, X.Y., Yu, X.J., Gil Wier, W., and Zang, W.J. (2013). Vagal stimulation triggers peripheral vascular protection through the cholinergic anti-inflammatory pathway in a rat model of myocardial ischemia/reperfusion. Basic Res Cardiol 108(3), 345. doi: 10.1007/s00395-013-0345-1.

**Supplemental. Figure.S1. VNS increased SDF-1α expression in the infarcted hearts, which could be abloshed by local injection of Ad-shSDF-1α into infracted hearts.**

To explore if VNS induced SDF-1α expression in the infarcted hearts, immunostaining staining for SDF-1α was used, and gray values were analyzed for semi-quantitative analysis of SDF-1α. And then, to confirm if VNS-induced SDF-1α involved in VSMCs changes, local injection of Ad-shSDF-1α into infracted hearts were used, and the knockdown efficiency of SDF-1α by shRNA 7 days after injection, were observed by immunostaining staining for SDF-1α.


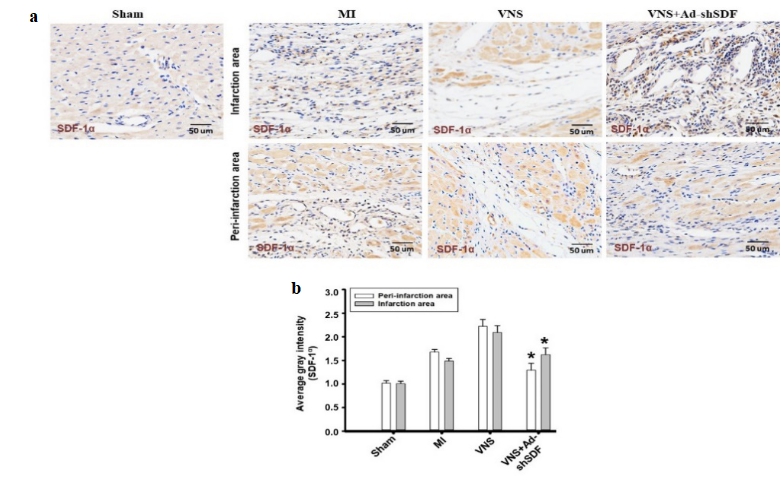


**Figure.S1. VNS increased SDF-1α expression in the infarcted hearts, which could be abloshed by local injection of Ad-shSDF-1α into infracted hearts.**

(a)Typical image for SDF-1α positive vessels in infracted hearts as determined by immunostaining of SDF-1α. (b) Quantitative analysis for SDF-1α levels in infracted hearts. ^#^*P＜*0.05 vs VNS.(n=6).

**Figure.S2. VNS decreased inflammatory response in myocardium infarction through m/n-AChR-SDF-1α**

To explore if VNS induced SDF-1α involved in inflammatory response in the infarcted hearts, Elisa for TNFα and IL-1β were used, and serumα-SMA-positive vessels of infracted hearts were analyzed.


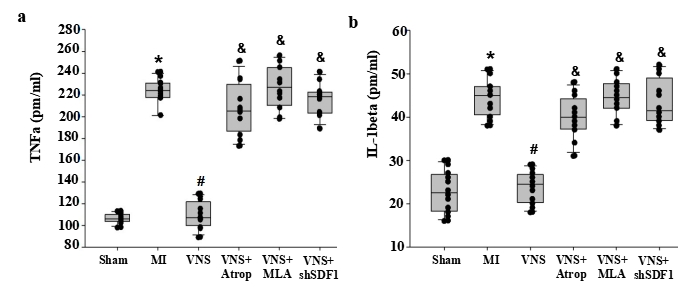


**Figure.S2.** **VNS decreased inflammatory response in myocardium infarction through m/n-AChR-SDF-1α**

(a-b) Elisa for TNFα and IL-1β in serum after the indicated treatment for MI. **P＜*0.05 vs Sham; ^#^*P＜*0.05 vs MI; ^&^*P＜*0.05 vs VNS (n=6).

**Figure.S3. VSMCs of MI hearts showed the expressions of m1~m5-AChR**

To confirm traits of m1~m5-AChR expression in VSMCs of MI hearts, immunohistofluorescencestaining for m1~m5-AChR were used, we found that VSMCs of MI hearts showed the expressions of m1~m5-AChR.


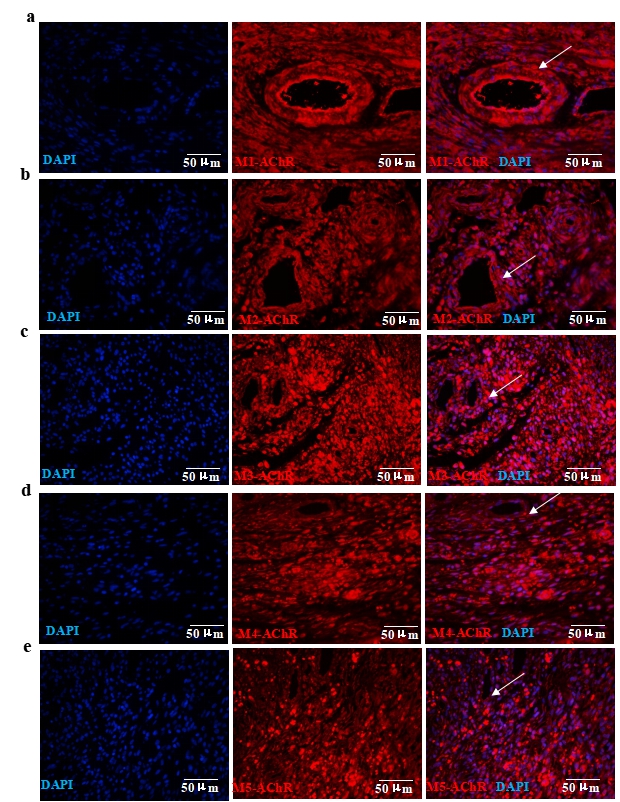
**Figure.S3. VSMCs of MI hearts showed the expressions of m1~m5-AChR**

(a-e)Typical immunohistofluorescence image of m1~m5-AChR in the infracted heart. Red color indicated the m1~m5-AChR; DAPI color indicated the stained nucleus. n=3.
