# Supplementary material for: VNS improves VSMC metabolism and arteriogenesis in infarcted hearts through m/n-AChR-Akt-SDF-1α in adult male rats
Source: J Mol Histol. 2024 Jan 2;55(1):51–67. doi: 10.1007/s10735-023-10171-4 (PMC10830782; doi:10.1007/s10735-023-10171-4)
Supplement: Supplementary file 2 — Supplementary material 2 (DOCX 2000.6 kb) [file 10735_2023_10171_MOESM2_ESM.docx]

**Supplemental Figure source results for**

**“VNS improves VSMC metabolism and arteriogenesis in infarcted hearts through m/n-AChR-Akt-SDF-1α in adult male rats”**

**Figure 9. ACh induced SDF-1α in VSMCs through m/n-AChR-Akt signaling pathway**

**(a-b)**ACh dose-dependently induced SDF-1α expression in VSMCs as detected by Western blot(a);(b) Semi quantitative analysis for SDF-1α expression in VSMCs. **P＜*0.05 vs 0 Mol/L ACh; ^#^*P＜*0.05 vs 10^-6^ Mol/L ACh (n=3).(c-e) The increased SDF-1α and phosphorylation of Akt following ACh stimulation in VSMCs was abolished by **mACh-R inhibitor a**tropine (Atrop) or nA**Ch-R inhibitor mecamylamine as** asdeternmined by Western blot (c); (d-e) Semi quantitative analysis for SDF-1α expression in VSMCs. **P＜*0.05 vs 0 Mol/L ACh; ^#^*P＜*0.05 vs 10^-5^ Mol/L ACh (n=3).

(f-g)Signaling pathways mediated the ACh-induced SDF-1α expression were assessed by pathway-specific inhibitors as indicated. Western blot was used to detect ACh-induced SDF-1αexpression in VSMCs following the treatment with Wortmannin (WM, 50 nM), LN (15nM), SB203580 (SB, 30 μM), PD98059 (PD, 50 μM), respectively. α-tubulin or β-actin served as an internal control; (g) Semi quantitative analysis of SDF-1αin figure 7F as indicated.At least three independent experiments were carried out. **P＜*0.05 vs. 0 ACh ( Ctrl); ^#^*P＜*0.05 vs. 10^-5^ Mol/L ACh; ^#^*P＜*0.05 vs. ACh+WM(n=3).

**Figure 9A for SDF-1**

Group:


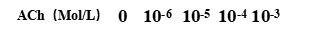


Three independent experiments for SDF-1α

**
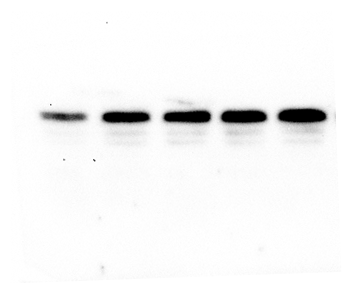
**

α-Tubulin

52 kDa

SDF-1α

11 kDa

**
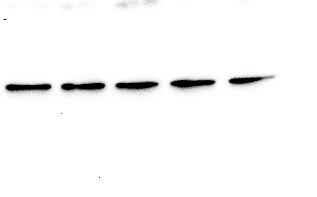
**


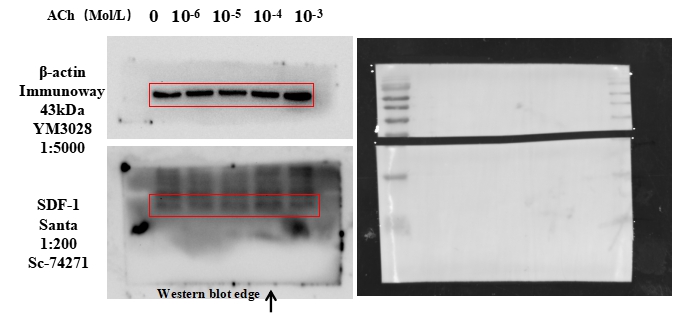


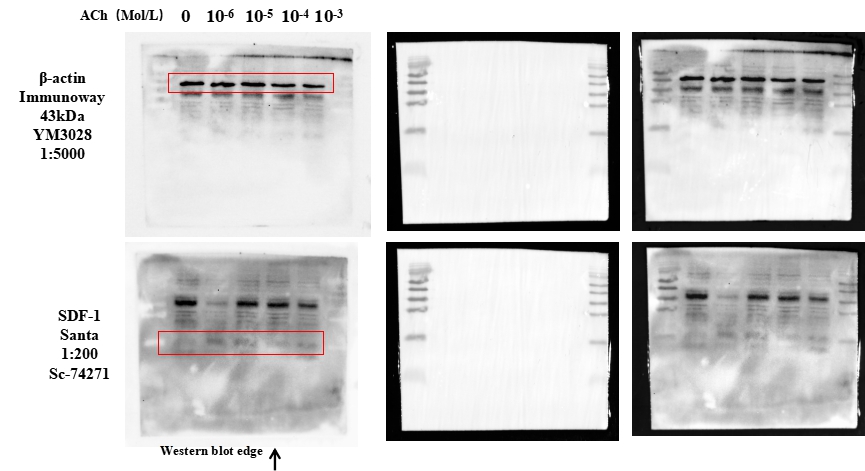


To generate this image, Lysates of myoblasts were analyzed at 6 days of differentiation. First, samples (20 μg proteins) were run on an 10 % SDS-PAGE gel then transferred onto a PVDF membrane (Millipore). Membranes were blocked in 5% nonfat milk in TBS-0.1 % Tween® 20 (TBS-T) before incubation with SDF-1α goat anti-rat (1:500, sc-6193, Santa Cruz, USA) or mouse anti-rat α-tubulin (T9026, 1:500, Sigma) antibody overnight at 4 ℃, respectively. Blots were washed four times in TBS-T, incubated with secondary antibodies for 90 min at room temperature, washed again four times then imaged. Secondary antibodies used were rabbit anti goat or goat anti-mouse IgG (1:10000, Jackson ImmunoResearch) preabsorbed at 1/2000 dilution.

Observed band size:SDF-1 α 11 kDa; α-tubulin 52 kDa

https://www.sigmaaldrich.cn/CN/zh/product/sigma/t9026

https://www.scbt.com/p/sdf-1-antibody-c-19?requestFrom=search

**Figure 9C for** SDF-1α and pAkt

Group:


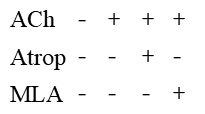


**
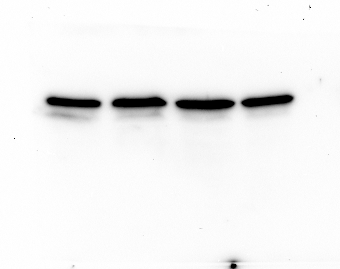
**

α-Tubulin

52 kDa

**
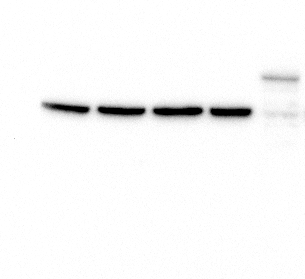
**

Akt

60 kDa

**
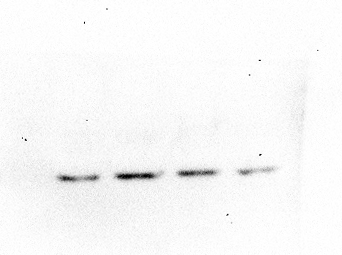
**

SDF-1α

11 kDa

**
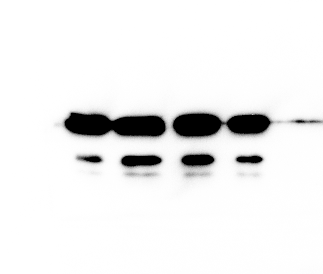
**

pAkt

60 kDa


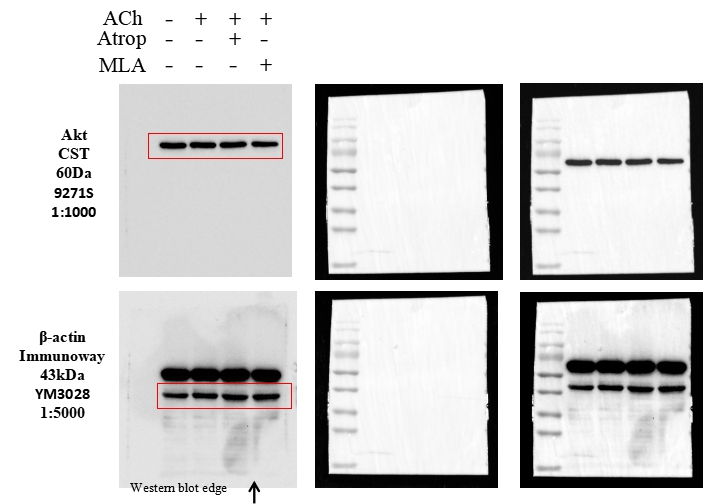


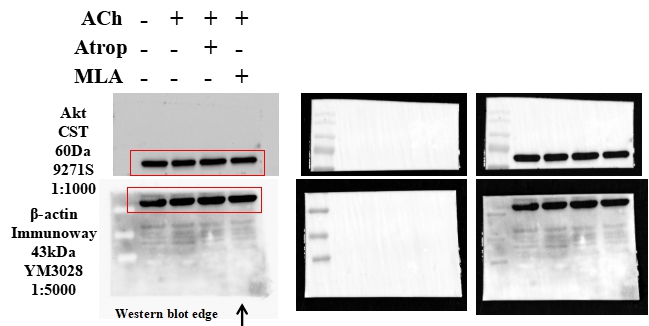


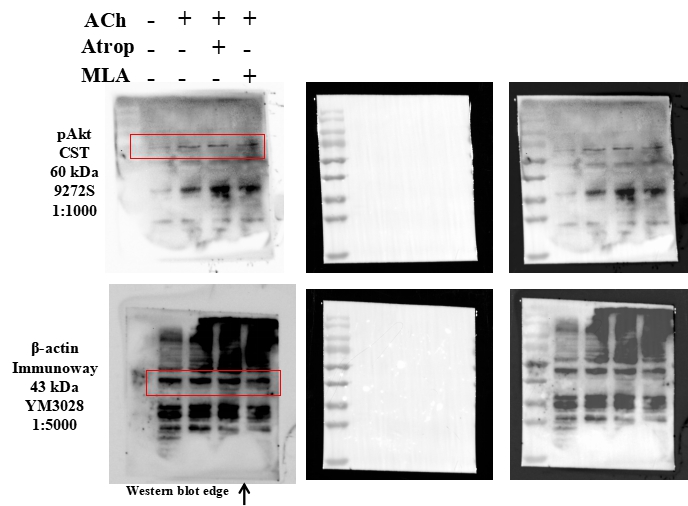


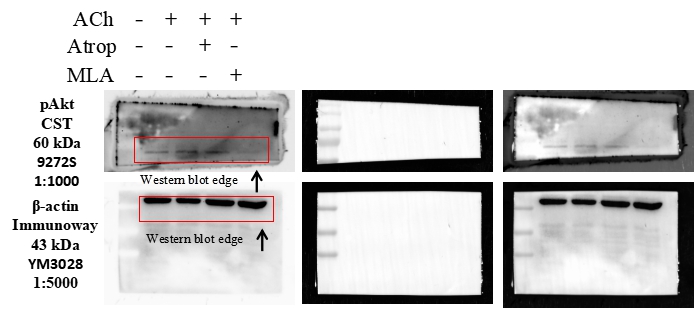


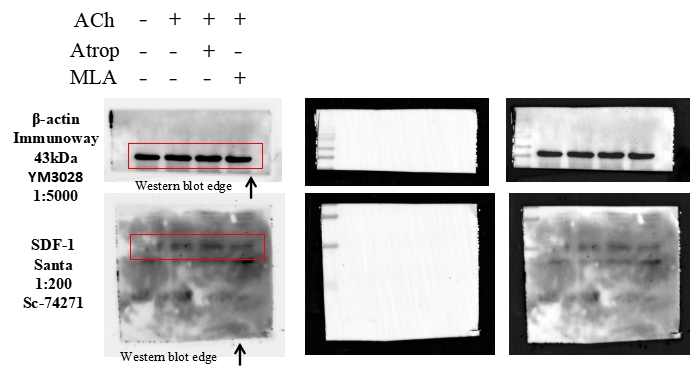


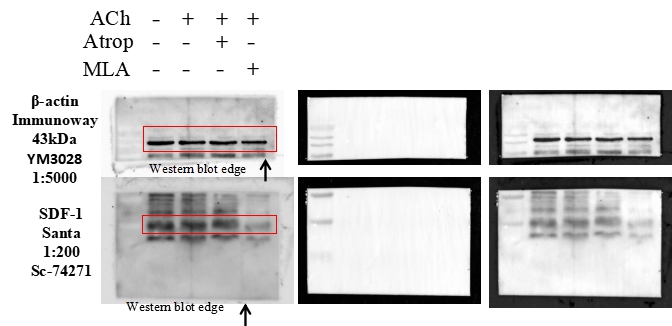


To generate this image, Lysates of myoblasts were analyzed at 6 days of differentiation. First, samples (20 μg proteins) were run on an 10 % SDS-PAGE gel then transferred onto a PVDF membrane (Millipore). Membranes were blocked in 5% nonfat milk in TBS-0.1 % Tween® 20 (TBS-T) before incubation with SDF-1α goat anti-rat (1:500, sc-6193, Santa Cruz, USA), rabbit anti-human Akt (9272S, 1:500; Cell Signaling), rabbit anti-human pAkt (9271S, 1:500; Cell Signaling) or mouse anti-rat α-tubulin (T9026, 1:500, Sigma) antibody overnight at 4 ℃, respectively. Blots were washed four times in TBS-T, incubated with secondary antibodies for 90 min at room temperature, washed again four times then imaged. Secondary antibodies used were goat anti-rabbit IgG or goat anti-mouse IgG (1:10000, Jackson ImmunoResearch)preabsorbed at 1/2000 dilution.

In order to save samples and workload, we divide the glue and film into two, one part was used to detect SDF-1 α, the other part was used to detect α-tubulin, AKT or pAKT. Unfortunately, the whole film and some films were not photographed.

Observed band size:SDF-1 α 11 kDa; α-tubulin 52 kDa; AKT:60kDa; pAKT:60kDa.

https://www.sigmaaldrich.cn/CN/zh/product/sigma/t9026

https://www.scbt.com/p/sdf-1-antibody-c-19?requestFrom=search

https://www.cellsignal.cn/products/primary-antibodies/akt-antibody/9272?site-search-type=Products&N=4294956287&Ntt=%239272&fromPage=plp&_requestid=96381

https://www.cellsignal.cn/products/primary-antibodies/phospho-akt-ser473-antibody/9271?site-search-type=Products&N=4294956287&Ntt=9271s&fromPage=plp&_requestid=96264

Figure 9F  **for** SDF-1α

Group:


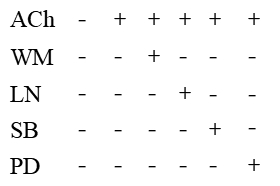


**
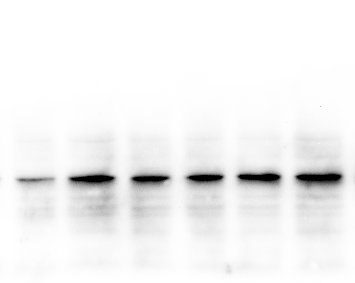
**

SDF-1α

11 kDa

**
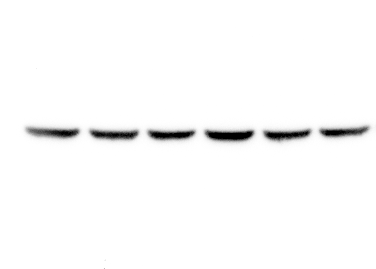
**

α-Tubulin

52 kDa


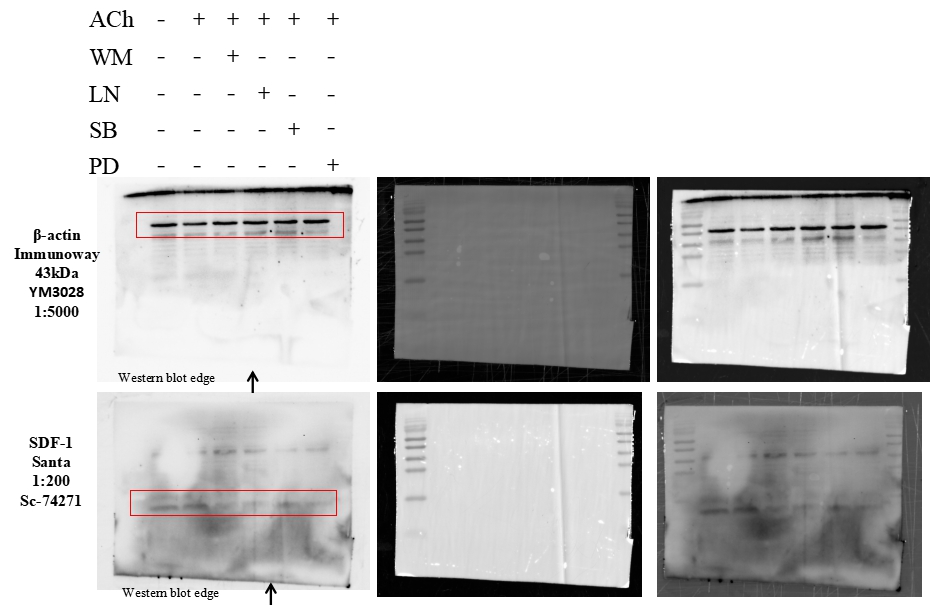


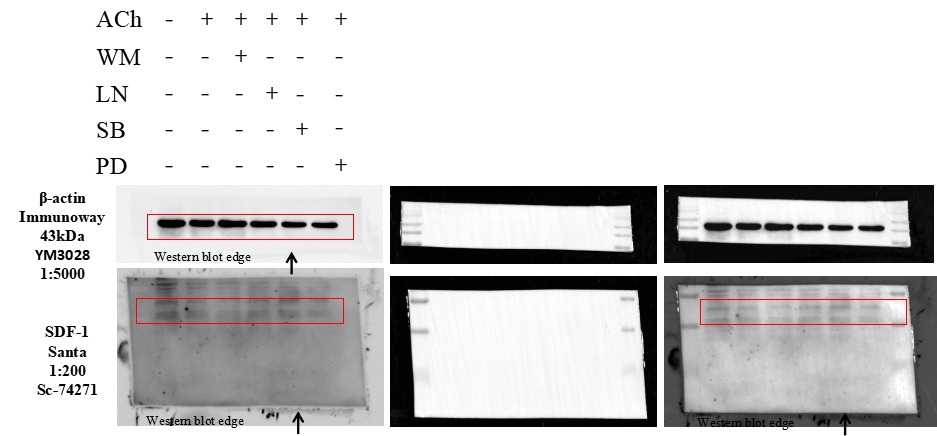


To generate this image, Lysates of myoblasts were analyzed at 6 days of differentiation. First, samples (20 μg proteins) were run on an 10 % SDS-PAGE gel then transferred onto a PVDF membrane (Millipore). Membranes were blocked in 5% nonfat milk in TBS-0.1 % Tween® 20 (TBS-T) before incubation with SDF-1α goat anti-rat (1:500, sc-6193, Santa Cruz, USA) or mouse anti-rat α-tubulin (T9026, 1:500, Sigma) antibody overnight at 4 ℃, respectively. Blots were washed four times in TBS-T, incubated with secondary antibodies for 90 min at room temperature, washed again four times then imaged. Secondary antibodies used were rabbit anti goat or goat anti-mouse IgG (1:10000, Jackson ImmunoResearch) preabsorbed at 1/2000 dilution.

Observed band size:SDF-1 α 11 kDa; α-tubulin 52 kDa

https://www.sigmaaldrich.cn/CN/zh/product/sigma/t9026

https://www.scbt.com/p/sdf-1-antibody-c-19?requestFrom=search
